# Reginald Horace Chilton

**Published:** 1902-06

**Authors:** 


### Obituary.

### REGINALD HORACE CHILTON.

The untimely death of Mr. Reginald Horace Chilton, at his residence in Clifton, has made an irreparable gap in a large circle of medical friends; especially will his loss be felt by those who have had the good fortune to work with him in hospital or under his guidance at the Medical School. At the latter institution he made himself much liked for the interest he took, not only in teaching, but also in the organisation of the Students' Club Union; in hospital the skill and care which he brought to bear upon his treatment of patients made him a valuable and interesting colleague.

During the discharge of his duties as Registrar to the Children's Hospital, to which post he had recently been appointed, he contracted diphtheria, and fell a victim to consequent heart failure.

Mr. Chilton was the third son of Mr. Horace Chilton, Deputy Sheriff of the City of Bristol. Having studied medicine at Bristol and Guy's, he obtained the conjoint qualification in March, 1893. From 1894 to 1896 he was House Surgeon at the Tiverton Infirmary, after which he went to Cook's School of Anatomy, where he was made Demonstrator of Anatomy prior to passing the first Fellowship Examination in November, 1896. After this he went to Egypt in charge of an invalid friend, and acted as medical officer at the Pilgrims' Camp at El Tor, on the Red Sea. During July, 1897, on his return to England, he was elected Junior House Physician to the Royal Infirmary, Bristol, and, six months later, Junior House Surgeon.

In 1899 he became Demonstrator of Physiology and Practical Histology at the Bristol Medical School.

Fourteen months before his death he started in general practice, and had held the Registrarship at the Children's Hospital for a few weeks only. For some months he had acted as Secretary to the Bristol branch of the St. John's Ambulance Association. Such popularity as his ensures a long-cherished memory.

